# A large-scale assessment of the quality of plant genome assemblies using the LTR assembly index

**DOI:** 10.1093/aobpla/plad015

**Published:** 2023-04-04

**Authors:** Morad M Mokhtar, Haytham M Abd-Elhalim, Achraf El Allali

**Affiliations:** African Genome Center, Mohammed VI Polytechnic University, Lot 660 Hay Moulay Rachid, Benguerir 43150, Morocco; Agricultural Genetic Engineering Research Institute, Agricultural Research Center, Giza 12619, Egypt; African Genome Center, Mohammed VI Polytechnic University, Lot 660 Hay Moulay Rachid, Benguerir 43150, Morocco

**Keywords:** assembled genomes, LTR assembly index, plant species

## Abstract

Recent advances in genome sequencing have led to an increase in the number of sequenced genomes. However, the presence of repetitive sequences complicates the assembly of plant genomes. The LTR assembly index (LAI) has recently been widely used to assess the quality of genome assembly, as a higher LAI is associated with a higher quality of assembly. Here, we assessed the quality of assembled genomes of 1664 plant and algal genomes using LAI and reported the results as data repository called PlantLAI (https://bioinformatics.um6p.ma/PlantLAI). A number of 55 117 586 pseudomolecules/scaffolds with a total length of 988.11 gigabase-pairs were examined using the LAI workflow. A total of 46 583 551 accurate LTR-RTs were discovered, including 2 263 188 *Copia*, 2 933 052 *Gypsy*, and 1 387 311 unknown superfamilies. Consequently, only 1136 plant genomes are suitable for LAI calculation, with values ranging from 0 to 31.59. Based on the quality classification system, 476 diploid genomes were classified as draft, 472 as reference, and 135 as gold genomes. We also provide a free webtool to calculate the LAI of newly assembled genomes and the ability to save the result in the repository. The data repository is designed to fill in the gaps in the reported LAI of existing genomes, while the webtool is designed to help researchers calculate the LAI of their newly sequenced genomes.

## Teaser

The LTR assembly index (LAI) has recently been widely used to assess the quality of genome assembly, as a higher LAI is associated with a higher quality of assembly. Here, we assessed the quality of the assembled genomes of 1664 land plant and algal genomes using the LAI values and reported the results in a data repository called PlantLAI (https://bioinformatics.um6p.ma/PlantLAI). We also provide a free web tool to calculate the LAI value of newly assembled plant genomes. The purpose of the data repository is to provide LAI values of existing genomes, while the web tool should allow researchers to calculate LAI values of their newly sequenced genomes.

## Introduction

The rapid development of plant whole-genome sequencing projects has been driven by next-generation sequencing technologies that offer extremely high throughput at affordable costs (Hunt *et al.* 2013). In recent decades, such projects have aided in producing superior crop varieties, uncover processes underpinning plant growth and development, and improve our knowledge of plant genome features such as complexity, size and architecture (Duitama *et al.* 2015; Cheng *et al.* 2016). However, current plant genome data derived from next-generation sequencing suffer from complications that make comprehensive chromosomal reconstruction difficult, such as read errors and large repeats in the genome (Mikheenko *et al.* 2018). Assessing the quality of genome assemblies is becoming increasingly important, both for assembly and reassembly and for the use of assembled genomes in downstream analysis (Yang *et al.* 2019). Because the precision of plant reference sequence data is critical to the interpretation of downstream functional genomic analysis, a measure of the quality of the genome sequences is needed.

Several methods have been developed to assess the quality of genome assemblies based on different concepts. These methods fall into two categories: length-based metrics and annotation-based metrics (Bradnam *et al.* 2013; Thrash *et al.* 2020). The N_50_ value is a length-based metric that represents the shortest fragment size at half the genome size (Ou *et al.* 2018). The N_50_ contig value is a commonly used method for evaluating the quality of assemblies, especially for determining the contiguity of assemblies. Contiguity is used to show how complete are assembled genomes and how many fragments or ‘contigs’ exist in the sequence. However, these values can be deceptive because the N_50_ value also increases when contigs are not assembled correctly. Moreover, they do not provide complete information about the completeness of genome assemblies (Gurevich *et al.* 2013; Hunt *et al.* 2013; Manchanda *et al.* 2020). The second category of metrics tests completeness by analyzing expected genome content, such as Benchmarking Universal Single-Copy Orthologs (BUSCO) and LTR Assembly Index (LAI; Simão *et al.* 2015; Ou *et al.* 2018; Waterhouse *et al.* 2018). BUSCO is commonly used to assess the absence or presence of multiple highly conserved orthologous genes (Simão *et al.* 2015). However, most recently assembled genomes as well as draft genomes have high BUSCO values, which is not sufficient to demonstrate genome completeness (Ou *et al.* 2018). While BUSCO can only assess gene space, the LTR Assembly Index (LAI) is efficient in estimating genome completeness in more repetitive genome regions by calculating the percentage of intact LTR retrotransposon (LTR-RT) sequences (Manchanda *et al.* 2020; Feron and Waterhouse 2022). Therefore, LAI is the efficient metric for analyzing plant genome assemblies that are often rich in repeats. Improvements in next-generation sequencing technologies for obtaining long-read genomes have recently led to a remarkable increase in the complete coverage of repetitive regions of plant genomes. Consequently, measurements from LAI have become extremely important (Ou *et al.* 2018).

The dynamics of whole-genome duplication and transposable elements (TE) are the main mechanisms responsible for the wide diversity of genome sizes in plants (Leebens-Mack *et al.* 2019). Within a given ploidy level, plant genome size and TE content are often linear (Lee and Kim 2014), so plant genome size varies mainly due to TEs, which makes plant genomes much more complicated than vertebrate genomes (Jiao and Schneeberger 2017). In some genomes, TE content ranges from 3 % to over 85 % and the genome size is positively correlated with the TE content (Kress *et al.* 2022). LTR-RTs are a class of TE scattered throughout most plant genomes and range in size from 4 to 20 kb (Mokhtar *et al.*2021a, 2023). Intact LTR-RT elements in the final plant genome assemblies yielded more intact elements than in the draft genomes, supporting the use of LAI as a measure of genome sequence quality and completeness (Ou *et al.* 2018).

Here we provide a large-scale assessment of the quality of the assembled genomes of 1664 plant and algal species using the LAI values and report the results in a data repository called PlantLAI, available at https://bioinformatics.um6p.ma/PlantLAI. We also provide a free web tool to calculate the LAI value of newly assembled plant genomes. The data repository is intended to fill in the gaps in the reported LAI values of existing genomes, while the web tool is intended to help researchers calculate the LAI values of their newly sequenced genomes. Today, our understanding of plant biology depends heavily on genomic data, for example, in identifying genomic regions that control plant–microbe interactions (Eid *et al.* 2021), exploring variations in gene expression under environmental stress (Omar *et al.* 2021), genome-wide identification studies (Atia *et al.* 2017; Ahmed *et al.* 2021) and clarifying the effects of genomic diversity among plant species (Mokhtar *et al.* 2021b). Providing a measure of plant genome quality will improve all downstream applications that rely on plant genome data.

## Materials and methods

A total of 1664 plant and algal genome sequences were retrieved from the NCBI database, including 1509 land plant genomes and 155 algal genomes. These genomes represent 704 plant species and 129 algal species. Of the 1509 land plants, 1456 are diploid genomes and 53 are polyploid genomes. For polyploid genomes, only the sequences assigned to one of their sub-genomes were analyzed. Some polyploids have more than one genome sequence in the NCBI database, here we only considered the sequences assigned to their sub-genomes. The species names, taxonomic groups, BioSample, BioProject, GenBank assembly accession, assembly level, genome size, and number of scaffolds/chromosomes of all genomes are provided in Supporting Information—Table S1.

**Figure 1.**
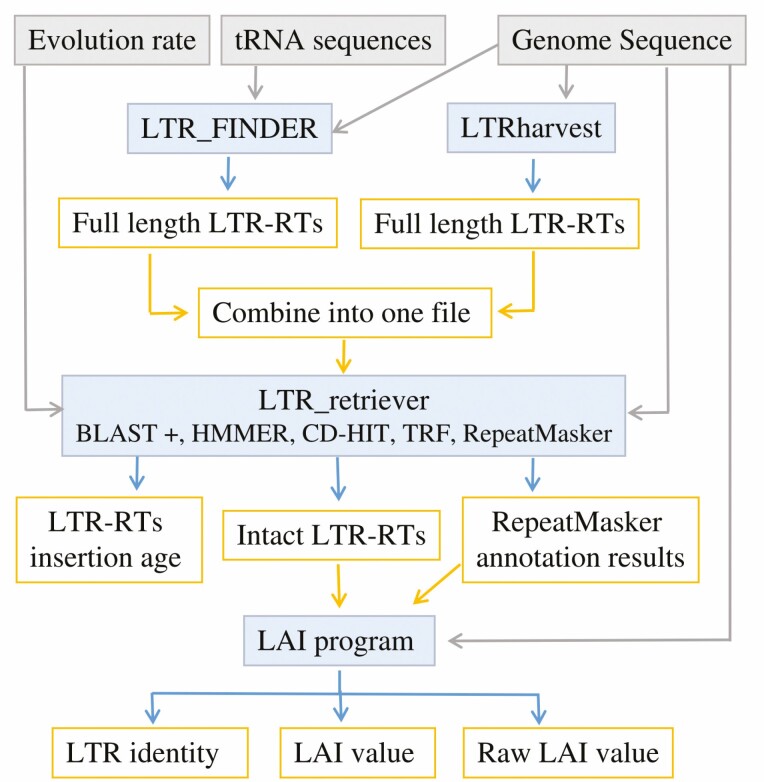
The workflow of LAI analysis, including the steps of identifying LTR-RTs, LTR identity, raw LAI and LAI value. The blue boxes represent the tools while the yellow boxes represent their output.

**Figure 2.**
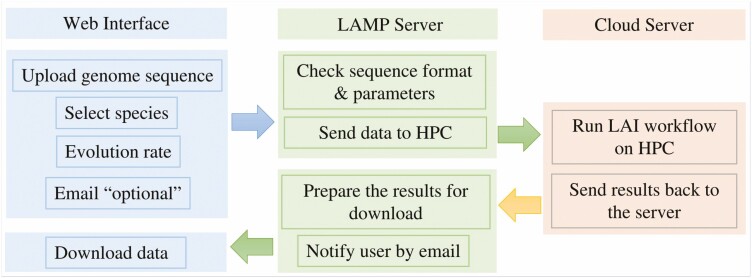
LAI online workflow, including the steps of receiving the data from the users, sending the data to the HPC, receiving the results and displaying them on the web interface.

The first step of the PlantLAI workflow is to detect LTR-RT candidates with LTRharvest (Ellinghaus *et al.* 2008), LTR_FINDER_parallel (Ou and Jiang 2019). The results are full-length LTR-RTs combined to detect the intact LTR-RTs with LTR_retriever which uses BLAST + (Camacho *et al.* 2009), HMMER (Wheeler and Eddy 2013), RepeatMasker (Smit *et al.* 2015), CD-HIT (Fu *et al.* 2012) and Tandem Repeats Finder (TRF) (Benson 1999). The intact LTR-RTs and the annotation of RepeatMasker are passed to the LAI program to compute the LAI values (Fig. 1). The LAI database and webserver use PHP 7.4.3, MongoDB 6.0, Apache 2.4 and Linux 5.4.0-89-generic x86 64. The server is powered by 16-core CPUs and has 32 GB RAM and a 10-TB hard disk. HTCondor 9.5 was used to manage and control the submitted tasks and jobs. The LAI online workflow includes the steps of receiving data from users, sending the data to the high-performance computer (HPC), receiving the results and preparing them for download through the web interface (Fig. 2). In-house scripts are used to transfer data between the server and the cloud. The jobs are queued and run on Toubkal (POWEREDGE C6420, CRC-STACKHPC, XEON PLATNIUM 8276L 28C 2.2GHZ, MELLANOX INFINIBAND HDR100), Africa’s fastest supercomputer as on August 2022 (Top 500.org 2022). LTRharvest (Ellinghaus *et al.* 2008), LTR_FINDER_parallel (Ou and Jiang 2019) and LTR_retriever (Ou and Jiang 2018) were used for LTR-RTs identification, while the LAI program (Ou *et al.* 2018) is used to estimate the LTR assembly index. The parameter settings for LTR_FINDER_parallel were as follows: *-seq genome -threads 56 -harvest_out -size 1000000 -time 300*. Parameter settings for LTR_FINDER (Xu and Wang 2007) in the parallel version were: *-w 2 -C -D 15000 -d 1000 -L 7000 -l 100 -p 20 -M 0.85 -s tRNA*. The tRNA sequences were retrieved from the plant transfer RNA database (Mokhtar and El Allali 2022). Parameters for LTRharvest_parallel were *-seq genome -threads 56 -gt genometools -size 1000000 -time 300*. For LTRharvest, the parameters in the parallel version were ‘*-minlenltr 100 -maxlenltr 7000 -mintsd 4 -maxtsd 6 -motif TGCA -motifmis 1 -similar 85 -vic 10 -seed 20 -seqids yes*’. LTR_retriever parameters were as follows: *-genome genome, -inharvest genome.rawLTR.scn* and *-threads 56*. For LTR assembly index, LAI beta3.2 (Ou *et al.* 2018) was used with the command line (*LAI -genome genome -intact genome.pass.list -all genome.out -t 56 -q -blast*) for diploid genomes. The parameter (*-mono chromosomes.ids*) is used for polyploid genomes.

## Results and Discussion

Recent advances in genome sequencing have led to an increase in the number of sequenced plant genomes (Mokhtar *et al.* 2021b). However, the presence of repetitive sequences complicates the assembly of a plant genome (Ou *et al.* 2018; Ou *et al.* 2019). Therefore, the growing number of assembled genomes in databases has increased the need to assess their quality (Ou *et al.* 2018). LAI was developed to assess the assembly of repetitive sequences and the completeness of assembly (Ou *et al.* 2018). To assess LAI of an assembled genome sequence, intact LTR-RTs and total LTR-RTs should represent at least 0.1 % and 5 % of the total genome size, respectively (Ou *et al.* 2018).

In the current study, to estimate the LAI value of 1664 land plant and algal genomes, a number of 55 117 586 pseudomolecules/scaffolds with a total length of 988.11 gb pairs were examined using LAI workflow (Fig. 1). For land plants, a total of 30 701 357 LTR-RT candidates were detected, including 6 583 551 LTR-RTs that passed the LTR_retriever filtering step and 24 117 806 false LTR-RTs elements. Only LTR-RT candidates that passed the LTR_retriever filtering step were used for further analysis. The 6 583 551 LTR-RT elements were divided into 2 263 188 *Copia*, 2 933 052 *Gypsy*, and 1 387 311 unknown superfamilies. For algae, a total of 229 348 LTR-RT candidates were detected, including 100 156 LTR-RTs that passed the LTR_retriever filtering step and 129 192 false LTR-RTs candidates. The 100 156 LTR-RT elements were divided into 39 666 *Copia*, 34 686 *Gypsy* and 25 804 unknown superfamilies. The details of the identified LTR-RTs elements, including the LTR-RTs that passed filtering and the intact LTR-RTs of each genome, have been deposited to PlantLAI (https://bioinformatics.um6p.ma/PlantLAI).

Consequently, 1136 plant genomes are suitable for calculating LAI values using the LAI program with values ranging from 0 to 31.59, and 373 genomes are not suitable for calculating LAI values. For algae, 36 genomes are suitable for calculating LAI values with values ranging from 0 to 44.33, and 120 genomes are not suitable for calculating LAI values [see Supporting Information—Table S2]. Because LAI can be used to identify low-quality genomic regions, the LAI and raw LAI values of pseudomolecules/scaffolds and their fragments (3 Mb) were estimated. Due to the large amount of data, the values for whole-genomes/pseudomolecules/scaffolds and their fragments for each genome can be retrieved from the PlantLAI search page.

**Figure 3.**
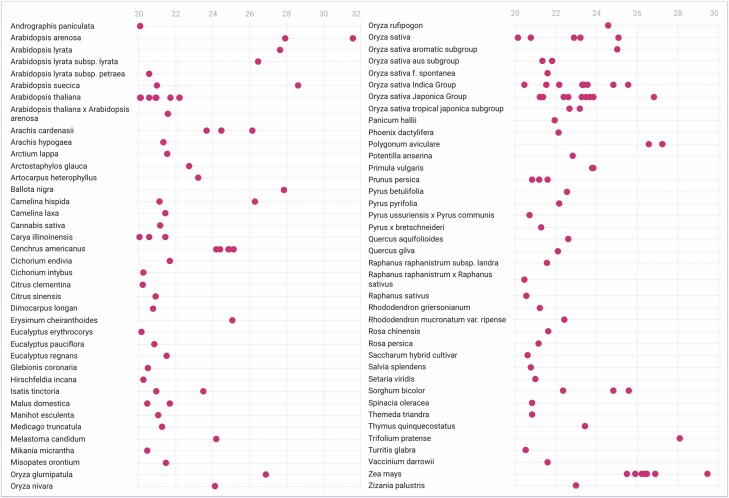
Dot plot of LAI values for diploid plant gold genomes. Genomes with more than one assembly version have multiple LAI values for each accession.

Ou *et al.* (2018) proposed a genome classification system for assembling repetitive and intergenic sequence space using the LAI value. They suggested that the LAI value for draft genomes is less than 10, whereas the LAI value for reference genomes is between 10 and 20, and gold-quality genomes have an LAI value greater than 20. Based on this quality classification system, diploid genomes were classified into 476 draft genomes, 472 reference genomes and 135 gold genomes, while polyploid sub-genomes were classified into 16 draft genomes, 98 reference genomes and 13 gold genomes. Polyploid genomes contain multiple sub-genomes that are not necessarily of the same quality ([see Supporting Information—Tables S2 and S3]; Fig. 3). Supporting Information—Figure S1 shows the histograms of the raw LAI and LAI values for the plant and algal genomes studied.

**Figure 4.**
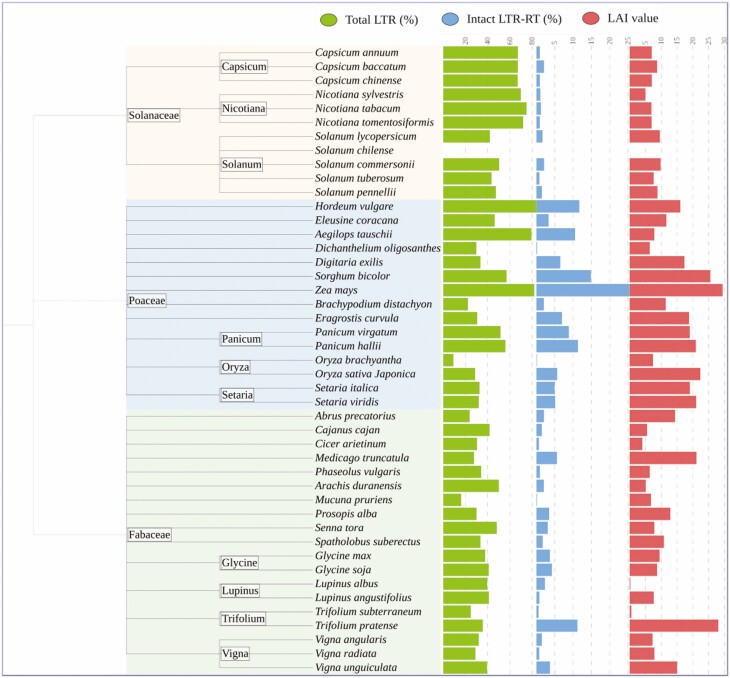
LAI value, percentage of total LTR and percentage of intact LTR-RT of Solanaceae, Poaceae and Fabaceae assembled genomes.

Considering NCBI reference genomes, 15 assembled genomes of cereal crops belonging to the Poaceae family are included in the analysis. The evaluation revealed that the genomes of *Zea mays* (GCA_902167145.1), *Sorghum bicolor* (GCA_000003195.3), *Oryza sativa* (GCA_001433935.1), *Setaria viridis* (GCA_005286985.1) and *Panicum hallii* (GCA_002211085.2) can be classified as gold genomes because their LAI values range from 21 to 29.45. Since the LAI values of *Aegilops tauschii* (GCA_002575655.2) and *Dichanthelium oligosanthes* (GCA_001633215.2) were 6.41 and 7.85, respectively, these genomes were classified as draft genomes, while the remaining genomes studied were considered as reference genomes. The *Hordeum vulgare* (GCA_904849725.1) and *Zea mays* genomes were the richest genomes in the Poaceae family in terms of their LTR-RTs content, accounting for 83.8 % and 81.9 % of the total genome, respectively (Fig. 4). Since more intact LTR-RTs are detected in these instances, higher LAI values are associated with higher quality assembly of intergenic and repetitive sequence regions of the genome (Ou *et al.* 2018; Takei *et al.* 2021). Recently, the LAI method has been widely used to assess the quality of genome assembly of cereal crops, such as rice (Yang *et al.* 2022), maize (Ou *et al.* 2020) and rye (Li *et al.* 2021). However, few reports examined the quality of the reference genomes on which the assembly of these genomes is based, and which are sometimes used for comparative analysis. PlantLAI fills the gap and provides LAI values of several versions of plant and algal genomes.

In addition, 19 NCBI reference genomes of legumes belonging to the Fabaceae family were evaluated. The analysis revealed that only the genomes of *Trifolium pratense* (GCA_020283565.1) and *Medicago truncatula* (GCA_003473485.2) could be classified as gold genomes, as their LAI values were 28.1 and 21.2, respectively. The genomes of *Vigna unguiculata* (GCA_004118075.2), *Abrus precatorius* (GCA_003935025.1), *Prosopis alba* (GCA_004799145.1) and *Spatholobus suberectus* (GCA_004329165.1) were classified as reference genomes, their LAI values ranged from 10.91 to 15.13, respectively. On the other hand, 13 genomes, including genomes of important legumes such as *Glycine max* (GCA_000004515.5), *Phaseolus vulgaris* (GCA_000499845.1), *Vigna radiata* (GCA_000741045.2) and *Cicer arietinum* (GCA_000331145.1), were classified as draft genomes. The genome of *Arachis duranensis* (GCA_000817695.3) was the richest genome of the Fabaceae family in terms of LTR-RTs and accounted for 50.6 % of the total genome (Fig. 4). Recently, Xi *et al.* (2022) used LAI to estimate the genome of *Vicia sativa* which was constructed using a mixture of long-reads from the Oxford Nanopore sequencing technology and short-reads from the Illumina sequencing technology. The LAI value was 12.96, which qualified the new genome to be considered as a reference genome.

The Solanaceae family contained 11 NCBI reference genomes among the evaluated genomes in this study, including three genomes from the genus *Nicotiana*, three genomes from the genus *Capsicum* and five genomes from the genus *Solanum*. The evaluation showed that all the NCBI reference genomes could be classified as draft genomes, as their LAI values ranged from 5.05 to 9.87. We could not perform the LAI analysis for the *Solanum chilense* (GCA_006013705.1) genome because the content of intact LTR-RT was 0.09 %, which is too low for accurate calculation of LAI. At least 0.1 % intact LTR-RT is required within the genome architecture. The genome of *Nicotiana tabacum* (GCA_000715135.1) was the richest genome of the Solanaceae family in terms of LTR-RTs, accounting for 75.07 % of the total genome (Fig. 4). Many previous studies have characterized LTR-RTs of Solanaceae family species such as *Capsicum annuum* (de Assis *et al.* 2020; Mokhtar *et al.* 2023), *Solanum melongena* (Barchi *et al.* 2019), *Datura stramonium* (De-la-Cruz *et al.* 2021) and *Solanum lycopersicum* (Paz *et al.* 2017; Mokhtar *et al.* 2023). In a recent study, the LAI method was used to verify the quality of *de novo* assembly of the genomes of wild tomatoes *Solanum pimpinellifolium* and *Solanum lycopersicum* var. *cerasiforme* which were sequenced with the PacBio Sequel system (Takei *et al.* 2021). The two genomes were categorized as reference because their LAI values were 14.18 and 13.10, respectively. In another recent study, Oxford Nanopore’s long-read sequencing technology was used to obtain the *de novo* genome sequence of a potato. The new genome was categorized as a reference genome because its LAI value was 13.56 (Pham *et al.* 2020).

### PlantLAI online data repository

Data generated by the LAI workflow of 1664 land plants and algal genomes were used to create an interactive web interface for LAI index. The Plant LAI (PlantLAI) website is accessible through the public link (https://bioinformatics.um6p.ma/PlantLAI). The PlantLAI data can be easily searched and downloaded from the website.

**Figure 5.**
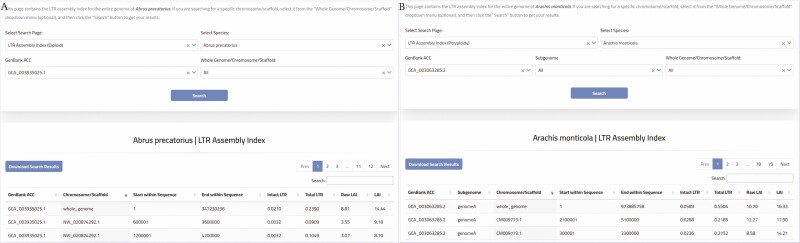
An example of PlantLAI Assembly Index search page for (A) Diploid, (B) Polyploid.

The PlantLAI search menu is divided into three individual search pages. The first page allows searching the LTR Assembly Index for selected diploid plant species. The second page allows searching for polyploid genomes, and the third page is designated for searching algal genomes. The LTR Assembly Index search page provides researchers with the LAI value for the entire genome of the selected species. In addition, a search option for a specific chromosome/scaffold is available. All results are displayed on the same page with information about LAI, including chromosome/scaffold, start and end of the analyzed sequence, percentage of intact LTR, percentage of total LTR, raw LAI, and LAI values (Fig. 5).

The download page can be accessed from the top menu of any page. The page provides option to download the bulk data as zipped files, including repeatmasker output, LTR-RTs passed by the LTR_retriever filter stage in bed format, the gff3 file for the identified intact LTR-RTs, the LAI table of the whole genome, pseudomolecules, scaffolds and their fragments, and the table of identified intact LTR-RTs.

### The LAI webserver

The web interface of the LAI pipeline tool is designed to retrieve the data required for the LAI workflow. The pipeline accepts whole-genome sequences in FASTA format. Users can upload genome file from a local computer or via an NCBI-FTP link. Users are also prompted to set the evolution rate and select the closest species for tRNA genes. The online LAI workflow consists of the identification of LTR-RT and the calculation of LAI using LTRharvest, LTR_FINDER, LTR_retriever and the LAI program (Figs 1 and 2).

The LAI webserver generates a series of files listing the LAI values, identified intact and non-intact LTR-RTs. The results table shows some key details such as LTR-RT superfamily, LTR-RT insertion age, percentage of intact LTR-RT, percentage of total LTR-RT, raw LAI and LAI values. The generated files include LTR-RTs identified by LTR_FINDER, LTRharvest and LTR_retriever in fasta, bed and gff3 formats. The output also includes all LTRs identified by the RepeatMasker software. The values of raw LAI and LAI for the whole genome, pseudomolecules, scaffolds and their fragments (3 Mb in size) are also made available for download and can be received by email if provided. The user can choose the option to save the results of the run, which will be added to the PlantLAI database after validation.

## Conclusion

The LAI method has recently been widely used to assess the quality of genome assembly after whole genome sequencing because a higher value of LAI is associated with higher quality assembly of the intergenic and repetitive sequence of the genome. Here, we assessed the quality of the assembled genomes of 1664 land plant and algal genomes using the LAI values and reported the results in a data repository called PlantLAI. We also provide a free web tool to calculate the LAI value of newly assembled plant genomes. The purpose of the data repository is to provide LAI values of existing genomes, while the web tool should allow researchers to calculate LAI values of their newly sequenced genomes. PlantLAI will be continuously updated with LAI values of newly deposited genomes as well as any updated reference genomes.

## Supporting Information

The following additional information is available in the online version of this article –

**Figure S1.** The histograms of the raw LAI and LAI values for the plant and algal genomes studied.

**Table S1.** The species name, taxonomy groups, GenBank assembly accession, BioSample id, BioProject id, Assembly level, genome size and number of scaffolds and chromosomes of all studied genomes.

**Table S2.** LAI values of all studied genomes

**Table S3.** The LAI values for 136 genomes with LAI values higher than 20 (gold quality)

plad015_suppl_Supplementary_Figure_S1

plad015_suppl_Supplementary_Table_S1

plad015_suppl_Supplementary_Table_S2

plad015_suppl_Supplementary_Table_S3

## Data Availability

All relevant data are within the manuscript, and Supporting information files. PlantLAI data repository and LAI webserver are freely available at https://bioinformatics.um6p.ma/PlantLAI

## Notes

**Phenome, Genome & Environment.Chief Editor:** Colleen Doherty

## References

[CIT0001] Ahmed SM , Alsamman AM, Jighly A, Mubarak MH, Al-Shamaa K, Istanbuli T, Momtaz OA, El Allali A, Hamwieh A. 2021. Genome-wide association analysis of chickpea germplasms differing for salinity tolerance based on DArTseq markers. PLoS One 16:e0260709. 34852014 10.1371/journal.pone.0260709 PMC8635330

[CIT0002] de Assis R , Baba VY, Cintra LA, Gonçalves LSA, Rodrigues R, Vanzela ALL. 2020. Genome relationships and LTR-retrotransposon diversity in three cultivated Capsicum L.(Solanaceae) species. BMC Genomics 21:1–14. 10.1186/s12864-020-6618-9 PMC7076952 32183698

[CIT0003] Atia MAM , Sakr MM, Mokhtar MM, Adawy SS. 2017. Development of sex-specific PCR-based markers in date palm. In: Al-Khayri J, Jain S, Johnson D, eds. Date Palm biotechnology protocols volume II: germplasm conservation and molecular breeding. New York, NY: Humana Press, 227–244.

[CIT0004] Barchi L , Pietrella M, Venturini L, Minio A, Toppino L, Acquadro A, Andolfo G, Aprea G, Avanzato C, Bassolino L, et al. 2019. A chromosome-anchored eggplant genome sequence reveals key events in Solanaceae evolution. Scientific Reports 9:1–13. 31409808 10.1038/s41598-019-47985-w PMC6692341

[CIT0005] Benson G. 1999. Tandem repeats finder: a program to analyze DNA sequences. Nucleic Acids Research 27:573–580. 9862982 10.1093/nar/27.2.573 PMC148217

[CIT0006] Bradnam KR , Fass JN, Alexandrov A, Baranay P, Bechner M, Birol I, Boisvert S, Chapman JA, Chapuis G, Chikhi R, et al. 2013. Assemblathon 2: evaluating de novo methods of genome assembly in three vertebrate species. GigaScience 2: 2047–217X. 10.1186/2047-217X-2-10 PMC3844414 23870653

[CIT0007] Camacho C , Coulouris G, Avagyan V, Ma N, Papadopoulos J, Bealer K, Madden TL. 2009. BLAST+: architecture and applications. BMC Bioinformatics 10:421. doi:10.1186/1471-2105-10-421 20003500 PMC2803857

[CIT0008] Cheng F , Wu J, Cai C, Fu L, Liang J, Borm T, Zhuang M, Zhang Y, Zhang F, Bonnema G, et al. 2016. Genome resequencing and comparative variome analysis in a Brassica rapa and Brassica oleracea collection. Scientific Data 3:1–9. 10.1038/sdata.2016.119 PMC5170593 27996963

[CIT0009] De-la-Cruz IM , Hallab A, Olivares-Pinto U, Tapia-López R, Velázquez-Márquez S, Piñero D, Oyama K, Usadel B, Núñez-Farfán J. 2021. Genomic signatures of the evolution of defence against its natural enemies in the poisonous and medicinal plant Datura stramonium (Solanaceae). Scientific Reports 11:1–19. 33441607 10.1038/s41598-020-79194-1 PMC7806989

[CIT0010] Duitama J , Silva A, Sanabria Y, Cruz DF, Quintero C, Ballen C, Lorieux M, Scheffler B, Farmer A, Torres E, et al. 2015. Whole genome sequencing of elite rice cultivars as a comprehensive information resource for marker assisted selection. PLoS One 10:e0124617. 25923345 10.1371/journal.pone.0124617 PMC4414565

[CIT0011] Eid AM , Fouda A, Abdel-Rahman MA, Salem SS, Elsaied A, Oelmüller R, Hijri M, Bhowmik A, Elkelish A, Hassan SE. 2021. Harnessing bacterial endophytes for promotion of plant growth and biotechnological applications: an overview. Plants 10:935. 34067154 10.3390/plants10050935 PMC8151188

[CIT0012] Ellinghaus D , Kurtz S, Willhoeft U. 2008. LTRharvest, an efficient and flexible software for de novo detection of LTR retrotransposons. BMC Bioinformatics 9:1–14. 18194517 10.1186/1471-2105-9-18 PMC2253517

[CIT0013] Feron R , Waterhouse RM. 2022. Assessing species coverage and assembly quality of rapidly accumulating sequenced genomes. GigaScience 11. doi:10.1093/gigascience/giac006 PMC8881204 35217859

[CIT0014] Fu L , Niu B, Zhu Z, Wu S, Li W. 2012. CD-HIT: accelerated for clustering the next-generation sequencing data. Bioinformatics 28:3150–3152. 23060610 10.1093/bioinformatics/bts565 PMC3516142

[CIT0015] Gurevich A , Saveliev V, Vyahhi N, Tesler G. 2013. QUAST: quality assessment tool for genome assemblies. Bioinformatics 29:1072–1075. 23422339 10.1093/bioinformatics/btt086 PMC3624806

[CIT0016] Hunt M , Kikuchi T, Sanders M, Newbold C, Berriman M, Otto TD. 2013. REAPR: a universal tool for genome assembly evaluation. Genome Biology 14:R471–R410. 10.1186/gb-2013-14-5-r47 PMC3798757 23710727

[CIT0017] Jiao W-B , Schneeberger K. 2017. The impact of third generation genomic technologies on plant genome assembly. Current Opinion in Plant Biology 36:64–70. 28231512 10.1016/j.pbi.2017.02.002

[CIT0018] Kress WJ , Soltis DE, Kersey PJ, Wegrzyn JL, Leebens-Mack JH, Gostel MR, Liu X, Soltis PS. 2022. Green plant genomes: What we know in an era of rapidly expanding opportunities. Proceedings of the National Academy of Sciences of the United States of America 119:e2115640118. 35042803 10.1073/pnas.2115640118 PMC8795535

[CIT0019] Lee S-I , Kim N-S. 2014. Transposable elements and genome size variations in plants. Genomics Informatics 12:87. 25317107 10.5808/GI.2014.12.3.87 PMC4196380

[CIT0020] Leebens-Mack JH , Wong GK; O. T. P. T. I. 2019. One thousand plant transcriptomes and the phylogenomics of green plants. Nature 574:679–685. 31645766 10.1038/s41586-019-1693-2 PMC6872490

[CIT0021] Li G , Wang L, Yang J, He H, Jin H, Li X, Ren T, Ren Z, Li F, Han X, et al. 2021. A high-quality genome assembly highlights rye genomic characteristics and agronomically important genes. Nature Genetics 53:574–584. 33737755 10.1038/s41588-021-00808-z PMC8035075

[CIT0022] Manchanda N , Portwood JL, Woodhouse MR, Seetharam AS, Lawrence-Dill CJ, Andorf CM, Hufford MB. 2020. GenomeQC: a quality assessment tool for genome assemblies and gene structure annotations. BMC Genomics 21:1–9. 10.1186/s12864-020-6568-2 PMC7053122 32122303

[CIT0023] Mikheenko A , Prjibelski A, Saveliev V, Antipov D, Gurevich A. 2018. Versatile genome assembly evaluation with QUAST-LG. Bioinformatics 34:i142–i150. 29949969 10.1093/bioinformatics/bty266 PMC6022658

[CIT0024] Mokhtar MM , El Allali A. 2022. PltRNAdb: Plant transfer RNA database. PLoS One 17:e0268904. 35605006 10.1371/journal.pone.0268904 PMC9126412

[CIT0025] Mokhtar MM , Alsamman AM, Abd-Elhalim HM, El Allali A. 2021a. CicerSpTEdb: A web-based database for high-resolution genome-wide identification of transposable elements in Cicer species. PLoS One 16:e0259540. 34762703 10.1371/journal.pone.0259540 PMC8584679

[CIT0026] Mokhtar MM , El Allali A, Hegazy M-EF, Atia MAM. 2021b. PlantPathMarks (PPMdb): an interactive hub for pathways-based markers in plant genomes. Scientific Reports 11:21300. 34716373 10.1038/s41598-021-00504-2 PMC8556342

[CIT0027] Mokhtar MM , Alsamman AM, El Allali A. 2023. PlantLTRdb: An interactive database for 195 plant species LTR-retrotransposons. Frontiers in Plant Science 14:1134627. 36950350 10.3389/fpls.2023.1134627 PMC10025401

[CIT0028] Omar SA , Fetyan NAH, Eldenary ME, Abdelfattah MH, Abd-Elhalim HM, Wrobel J, Kalaji HM. 2021. Alteration in expression level of some growth and stress-related genes after rhizobacteria inoculation to alleviate drought tolerance in sensitive rice genotype. Chemical and Biological Technologies in Agriculture 8:1–19.

[CIT0029] Ou S , Jiang N. 2018. LTR_retriever: a highly accurate and sensitive program for identification of long terminal repeat retrotransposons. Plant Physiology 176:1410–1422. 29233850 10.1104/pp.17.01310 PMC5813529

[CIT0030] Ou S , Jiang N. 2019. LTR_FINDER_parallel: parallelization of LTR_FINDER enabling rapid identification of long terminal repeat retrotransposons. Mobile DNA 10:1–3. 31857828 10.1186/s13100-019-0193-0 PMC6909508

[CIT0031] Ou S , Chen J, Jiang N. 2018. Assessing genome assembly quality using the LTR Assembly Index (LAI). Nucleic Acids Research 46:e126–e126. 30107434 10.1093/nar/gky730 PMC6265445

[CIT0032] Ou S , Su W, Liao Y, Chougule K, Agda JR, Hellinga AJ, Lugo CS, Elliott TA, Ware D, Peterson T, et al. 2019. Benchmarking transposable element annotation methods for creation of a streamlined, comprehensive pipeline. Genome Biology 20: 275. 31843001 10.1186/s13059-019-1905-y PMC6913007

[CIT0033] Ou S , Liu J, Chougule KM, Fungtammasan A, Seetharam AS, Stein JC, Llaca V, Manchanda N, Gilbert AM, Wei S, et al. 2020. Effect of sequence depth and length in long-read assembly of the maize inbred NC358. Nature Communications 11:1–10. 10.1038/s41467-020-16037-7 PMC7211024 32385271

[CIT0034] Paz RC , Kozaczek ME, Rosli HG, Andino NP, Sanchez-Puerta MV. 2017. Diversity, distribution and dynamics of full-length Copia and Gypsy LTR retroelements in Solanum lycopersicum. Genetica 145:417–430. 28776161 10.1007/s10709-017-9977-7

[CIT0035] Pham GM , Hamilton JP, Wood JC, Burke JT, Zhao H, Vaillancourt B, Ou S, Jiang J, Robin Buell C. 2020. Construction of a chromosome-scale long-read reference genome assembly for potato. GigaScience 9:giaa100. 32964225 10.1093/gigascience/giaa100 PMC7509475

[CIT0036] Simão FA , Waterhouse RM, Ioannidis P, Kriventseva EV, Zdobnov EM. 2015. BUSCO: assessing genome assembly and annotation completeness with single-copy orthologs. Bioinformatics 31:3210–3212. 26059717 10.1093/bioinformatics/btv351

[CIT0037] Smit AFA , Hubley R, Green P. RepeatMasker Open-4.0. 2013–2015. 2015. https://www.repeatmasker.org.

[CIT0038] Takei H , Shirasawa K, Kuwabara K, Toyoda A, Matsuzawa Y, Iioka S, Ariizumi T. 2021. De novo genome assembly of two tomato ancestors, Solanum pimpinellifolium and Solanum lycopersicum var. cerasiforme, by long-read sequencing. DNA Research 28:dsaa029. 33475141 10.1093/dnares/dsaa029 PMC7934570

[CIT0039] Thrash A , Hoffmann F, Perkins, A. 2020. Toward a more holistic method of genome assembly assessment. BMC Bioinformatics 21:1–8. 32631298 10.1186/s12859-020-3382-4 PMC7336394

[CIT0040] Top 500.org. 2022. https://www.top500.org/system/179908/.

[CIT0041] Waterhouse RM , Seppey M, Simão FA, Manni M, Ioannidis P, Klioutchnikov G, Kriventseva EV, Zdobnov EM. 2018. BUSCO applications from quality assessments to gene prediction and phylogenomics. Molecular Biology and Evolution 35:543–548. 29220515 10.1093/molbev/msx319 PMC5850278

[CIT0042] Wheeler TJ , Eddy SR. 2013. nhmmer: DNA homology search with profile HMMs. Bioinformatics 29:2487–2489. 23842809 10.1093/bioinformatics/btt403 PMC3777106

[CIT0043] Xi H , Nguyen V, Ward C, Liu Z, Searle IR. 2022. Chromosome-level assembly of the common vetch (Vicia sativa) reference genome. Gigabyte 2022:1–20. 10.46471/gigabyte.38 PMC9650280 36824524

[CIT0044] Xu Z , Wang H. 2007. LTR_FINDER: an efficient tool for the prediction of full-length LTR retrotransposons. Nucleic Acids Research 35:W265–W268. 17485477 10.1093/nar/gkm286 PMC1933203

[CIT0045] Yang L-A , Chang Y-J, Chen S-H, Lin C-Y, Ho J-M. 2019. SQUAT: a Sequencing Quality Assessment Tool for data quality assessments of genome assemblies. BMC Genomics 19:1–12. 10.1186/s12864-019-5445-3 PMC7402383 30999844

[CIT0046] Yang L , Zhao M, Sha G, Sun Q, Gong Q, Yang Q, Xie K, Yuan M, Mortimer JC, Xie W, et al. 2022. The genome of the rice variety LTH provides insight into its universal susceptibility mechanism to worldwide rice blast fungal strains. Computational and Structural Biotechnology Journal 20:1012–1026. 35242291 10.1016/j.csbj.2022.01.030 PMC8866493

