# Supplementary figures and images for: A large-scale assessment of the quality of plant genome assemblies using the LTR assembly index
Source: AoB Plants. 2023 Apr 4;15(3):plad015. doi: 10.1093/aobpla/plad015 (PMC10184434; doi:10.1093/aobpla/plad015)

**Figure S1.** The histograms of the raw LAI and LAI values for the plant and algal genomes studied.


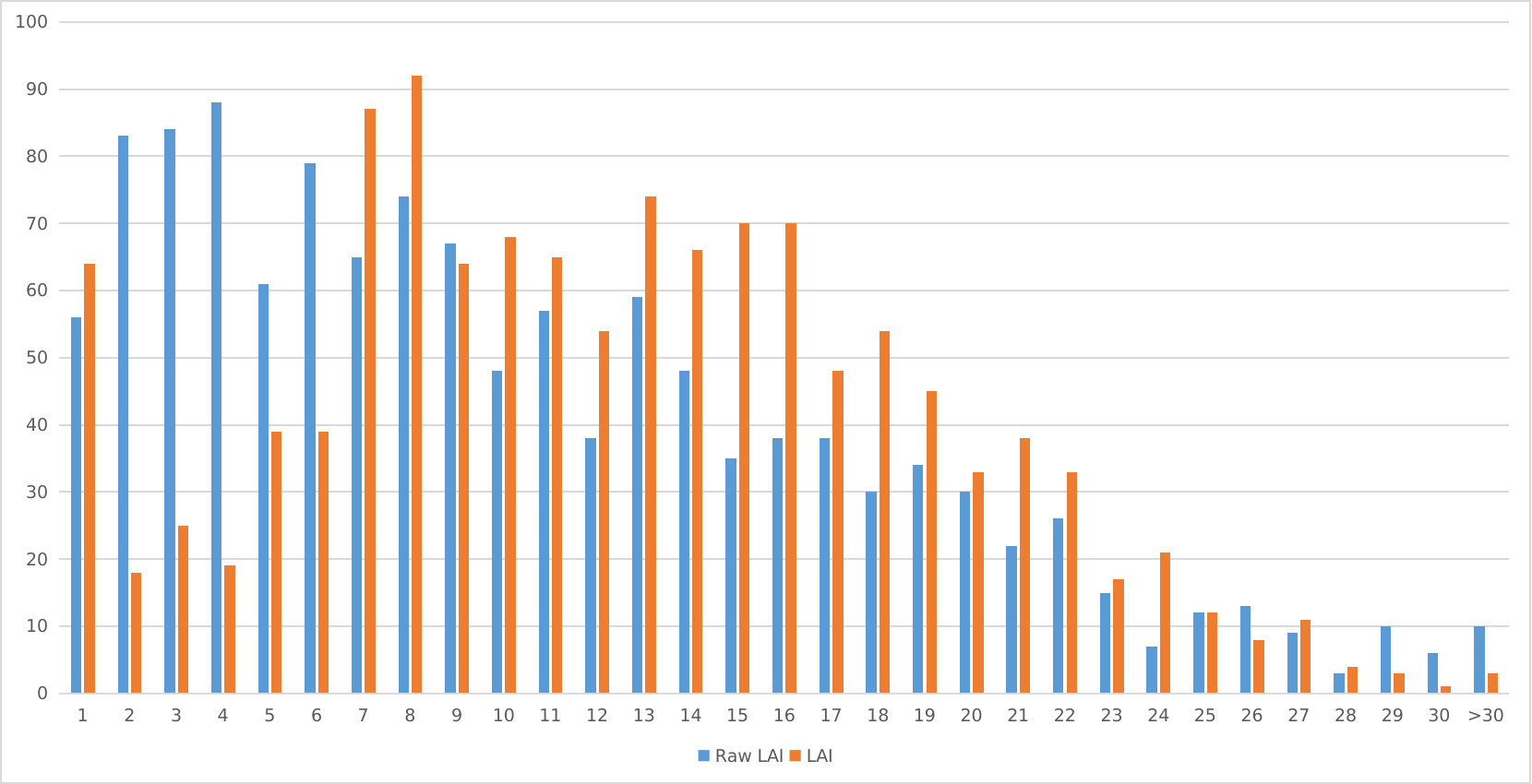

Supplement: plad015_suppl_Supplementary_Figure_S1 [file plad015_suppl_supplementary_figure_s1.docx]
